# Patient neglect in healthcare institutions: a systematic review and conceptual model

**DOI:** 10.1186/1472-6963-13-156

**Published:** 2013-04-30

**Authors:** Tom W Reader, Alex Gillespie

**Affiliations:** 1 Institute of Social Psychology, London School of Economics, Houghton Street, London WC2A 2AE, UK

**Keywords:** Neglect, Patient safety, Caring, Organisational culture, Systematic review

## Abstract

**Background:**

Patient neglect is an issue of increasing public concern in Europe and North America, yet remains poorly understood. This is the first systematic review on the nature, frequency and causes of patient neglect as distinct from patient safety topics such as medical error.

**Method:**

The Pubmed, Science Direct, and Medline databases were searched in order to identify research studies investigating patient neglect. Ten articles and four government reports met the inclusion criteria of reporting primary data on the occurrence or causes of patient neglect. Qualitative and quantitative data extraction investigated (1) the definition of patient neglect, (2) the forms of behaviour associated with neglect, (3) the reported frequency of neglect, and (4) the causes of neglect.

**Results:**

Patient neglect is found to have two aspects. First, procedure neglect, which refers to failures of healthcare staff to achieve objective standards of care. Second, caring neglect, which refers to behaviours that lead patients and observers to believe that staff have uncaring attitudes. The perceived frequency of neglectful behaviour varies by observer. Patients and their family members are more likely to report neglect than healthcare staff, and nurses are more likely to report on the neglectful behaviours of other nurses than on their own behaviour. The causes of patient neglect frequently relate to organisational factors (e.g. high workloads that constrain the behaviours of healthcare staff, burnout), and the relationship between carers and patients.

**Conclusion:**

A social psychology-based conceptual model is developed to explain the occurrence and nature of patient neglect. This model will facilitate investigations of i) differences between patients and healthcare staff in how they perceive neglect, ii) the association with patient neglect and health outcomes, iii) the relative importance of system and organisational factors in causing neglect, and iv) the design of interventions and health policy to reduce patient neglect.

## Background

Patient neglect, defined as “the failure of a designated care giver to meet the needs of a dependent” [1] (p.437), has become an issue of concern in both North America and Europe [2,3]. In the UK, this has been driven by media outlets [4,5], charities [6], and health regulators [7]. Headlines such as “*Want to know the NHS’s real problem? Ask a nurse for a bowl of cornflakes*” [8], “*Shamed hospital accused of leaving dying patients to starve*” [9], and “*Can patient neglect be a violation of human rights*?” [10] capture concerns relating to patient neglect. They reflect public anxiety, with patients and families making 22,845 complaints to the NHS in 2011 on issues relating to staff attitudes, communication, and patient dignity [11]. Senior politicians acknowledge the issue, and argue that neglect has been “*hidden away*” [12] and that healthcare institutions must ensure *“every patient is cared for with compassion and dignity*” [13]. Solutions include “*reducing stifling bureaucracy*” [14], ensuring nursing staff talk to patients at least “*once an hour*” [13], utilising legislation and regulation to ensure staff consider patient’ “*wellbeing and dignity*” [15], and making staff sign-up to a “*code of conduct*” on dignity and respect [16]. The solutions reflect a belief that healthcare staff are responsible for instances of patient neglect, but they are also contradictory (e.g. reducing bureaucracy to free staff from form-filling whilst simultaneously increasing bureaucracy to ensure staff care for patients properly), or involve regulating aspects of behaviour that are difficult to measure and assumed to be lacking (e.g. compassion). These contradictions reveal the lack of a clear understanding of the nature and causes of patient neglect.

High-profile scandals have made patient neglect a key issue for policy makers. Scandals have included patients being regularly physically (e.g. left malnourished, dehydrated, in pain, and unwashed) or emotionally (e.g. being ignored whilst in need, not shown compassion, loss of dignity) neglected by healthcare staff [17-20]. Linking patient neglect to specific metrics of patient harm or clinical outcomes is difficult due to the often complex conditions of patients and their treatment [21]. Furthermore, conducting research is challenging due to the toxicity of the subject (e.g. questioning the abilities, motivation and ethics of staff) and a media narrative which seeks to blame rather than understand why poor care occurs [22,23]. However, cases such as the Mid-Staffordshire NHS Foundation Trust scandal, where routine and basic failings in care resulted in up to 1,200 patients deaths between 2005 and 2008, show the catastrophic implications for patient care when neglect becomes systemic across an organisation [21,24].

Researchers in medicine, health sciences, and psychology have for some time investigated how institutional processes, clinical environments, and the behaviour of healthcare staff influence patient safety [25]. These investigations have resulted in interventions (e.g. team-training, care bundles, skill validation) to reduce medical error and improve clinical outcomes [26]. Although they might be expected to reduce patient neglect, it appears necessary for practical (e.g. to meet public and political concerns) and conceptual reasons (e.g. to develop suitable interventions) to distinguish patient neglect from unintentional error, or intentional abuse. This is because reports on neglect such as those cited above often refer to: i) staff behaviours that may not directly lead to patient harm (e.g. not aiding patients to go to the toilet), but are crucial for care and probably do not reflect a competency gap; ii) staff attitudes and behaviours towards patients that cannot be regulated or easily measured (e.g. compassion); iii) a mixture of causal factors leading to patient neglect, some of which indicate neglect to be unintentional (e.g. due to a lack of resources) or alternatively not related to error (e.g. rudeness) [27]; iv) differing beliefs between patients, families, and staff as to whether neglect has occurred (e.g. for loss of patient dignity) and the causes of neglect; and v) breakdowns in institutional structures (e.g. communication between staff and management) that are a prerequisite to introducing interventions to improve care [26].

This article reviews the research literature on patient neglect, and interprets this work within the framework of organisational and social psychology. This structure is utilised in order to reflect the observation that patient neglect emerges from a complex mixture of organisational (e.g. resources, management) and social factors (e.g. relationships between patients and healthcare staff). In particular, the interactions and perspectives of staff and patients appear especially important for understanding when and why neglect occurs. The overall aim of the review is to contribute to the public dialogue and academic understanding of neglect. Its specific objectives are to:

1) Review what is meant by patient neglect, and consider how it differs from other constructs relating to poor patient care.

2) Describe the staff behaviours reported in studies of patient neglect.

3) Examine how healthcare staff and patients perceive neglect (and whether there are differences).

4) Identify the causal factors commonly cited as leading to instances of patient neglect.

## Method

**Figure 1:**
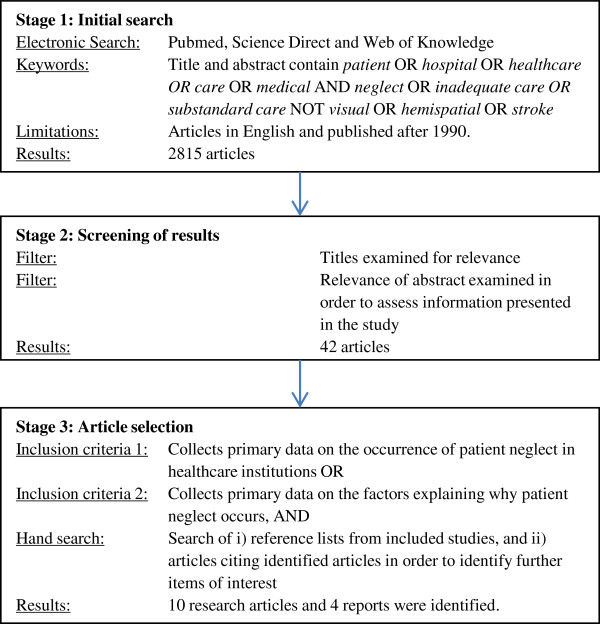
Literature review flow diagram.

This is the first literature review on the nature and causes of patient neglect. Accordingly no protocol exists to guide the review, so standard protocols for literature review were applied [28]. The eligibility criteria were articles or reports published in English reporting primary data, since 1990, on the occurrence or causes of patient neglect anywhere in the world. In the first instance, the search for articles on patient neglect was framed using Lachs and Pillemer’s [1] (p.437), widely used definition (in reference to neglect of elderly patients) of “the failure of a designated care giver to meet the needs of a dependent”. From this perspective, patient neglect is behavioural (intentionally or unintentionally failing to meet the needs of a caregiver). The information sources, search terms used, and study selection procedure are outlined in Figure 1.

To evaluate the methodological quality of the research studies, we applied the SIGN system [29]. This provides ratings through which to assess the quality of data collected in quantitative and qualitative studies. The assessments for each study are reported in Table 1, with the quality ratings being the following:

1++: High quality meta-analyses, systematic reviews of RCTs, or RCTs with a very low risk of bias.

1+: Well-conducted meta-analyses, systematic reviews, or RCTs with a low risk of bias.

1-: Meta-analyses, systematic reviews, or RCTs with a high risk of bias.

2++: High quality systematic reviews of case control or cohort or studies. High quality case control or cohort studies with a very low risk of confounding or bias and a high probability that the relationship is causal.

2+: Well-conducted case control or cohort studies with a low risk of confounding or bias and a moderate probability that the relationship is causal.

2-: Case control or cohort studies with a high risk of confounding or bias and a significant risk that the relationship is not causal.

3: Non-analytic studies, e.g. case reports, case series.

4: Expert opinion.

**Table 1:** Behaviours and causal factors identified in studies of patient neglect

**Author**	**Method, setting, and participants**	**SIGN quality rating**	**Procedure neglect (failings in care that fall short of objective and observable institutional and professional standards)**	**Caring neglect (failings in care that are below the threshold of being proceduralised, yet lead patients, family and the public to believe that staff are unconcerned about the emotional and physical wellbeing of patients)**	**Proximal (e.g. on-the-ground) causes of neglect**	**Distal (e.g. institutional) causes of neglect**
1. Jewkes et al., 1998 [41]	103 qualitative interviews with patients and nursing staff at two South African obstetric units. Interviews focussed on treatment of patients by nursing staff	3	Not attending to patient hygiene	Rudeness to patients; Not responding to patient requests or complaints of pain; Not attending to patient comfort; Talking to staff rather than patients	High workloads and low staffing levels; Problems in relationship between staff and patients	Normalisation of neglect; Failure of management to ensure quality of care
2. Goergen, 2001 [32]	Survey of 80 staff in nine German nursing homes Measured if staff had engaged or observed patient neglect in the previous 12 months	2-	Not changing patient linen or underwear (8% committed, 18% observed ); Not washing patients (11% committed, 12% observed); Not changing bedridden patient’s position (31% committed, 30% observed)	Ignoring patients (35% committed, 31% observed); Delaying help for patients (19% committed, 26% observed)	High workloads and low staffing levels; Patient’ ill-treatment of nursing staff; Staff stress and burnout	Low staff morale
3. Erikkson & Saveman, 2002 [40]	Qualitative interviews with 12 nurses who cared for patients with dementia in acute care settings	3		Not meeting patient needs; Ignoring patients; Responding to preferred patients over others	High workload; Low staffing levels; Difficulties in communicating with patients; Poor teamwork	Organisational change and instability; Poor technical environment
4. Georgen, 2004 [39]	Qualitative interviews (e.g. patients, nursing staff, families) with 251 participants in eight nursing homes, and survey of 361 nursing care home staff across 27 institutions	2-	Not changing bedridden residents position in time (29% observed); Neglecting resident’s oral hygiene (28% observed)	Psychosocial neglect (29% committed, 34% observed); Intentionally ignoring resident (26% observed)	Ratio of staff to patients; Staff burnout; Staff shortages, time pressure and workload	Organisational politics; Culture for patients voicing concern; Low staff morale Satisfaction with management
5. Wang, 2005 [38]	Cross-sectional survey of 114 caregivers in long-term care facilities in Taiwan. Respondents completed the ‘caregiver psychological abuse behaviours’ scale	2-		Not responding to requests for help; Not listening to patients	High workloads	Low levels of staff education; Lack of appropriate training for staff
6. Swahnberg et al., 2006 [36]	Survey of 661 obstetric patients in Sweden. Measured patient experiences of medical staff ‘disobeying ethical principles’	2-		Ignoring patients (79% experienced); Not acknowledging patient opinions (27% experienced); Not giving patients enough time to consider treatments (25% experienced); Excluding patients from decision-making (20% experienced)		Culture for accepting ethical codes of practice
7. Khalil., 2009 [34]	Qualitative survey of 373 nurses on behaviours and attitudes towards ‘good’ and ‘bad’ patients in eight South African public hospitals	2-		Ignoring patients; Avoiding contact with patients; Not providing adequate care	High workload and shortage of staff; Categorisation of patients as ‘bad’ or problematic	Low staff morale
8. Malmedal et al., 2009 [35]	Survey of 616 nursing staff in 16 Norwegian nursing homes Survey measured how often staff had engaged or observed colleagues neglecting patients once a week or less	2-	Neglecting oral care (9% committed, 17% observed); Not providing appropriate nutrition (0.3% committed, 3% observed); Not washing patients (15% committed, 7% observed); Not treating an injury/wound adequately (0.3% committed, 3% observed)	Ignoring a patient (5% committed, 15% observed); Delaying care assistance longer than necessary (4% committed, 15% observed)	Staff burnout and stress	Education and training
9. UK Parliamentary and Health Ombudsman., 2009 [44]	Qualitative investigations (interviews and document analyses) into the death of six NHS patients with learning difficulties	3	Not providing appropriate nutrition or hydration; Not providing appropriate provide pain relief; Not appropriately considering patient readiness for discharge; Not appropriately planning pain management; Failures to observe, monitor, or record patient data.	Not responding to concerns/complaints from families; Failure to communicate with patients and families; Lack of empathy for patients	Protocol breaches; Errors in noticing deficiencies in patient care	Administrative/organisational errors; Lack of organisational leadership on priorities of care; Lack of appropriate training for staff
10. Entwistle et al., 2010 [42]	Qualitative study with 71 interviews and 12 focus groups investigating negative staff reactions to speaking up on patient safety	3		Not responding to concerns/complaints from families	Problems in relationship between staff and patients	Poor safety culture
11. Francis, 2010 [24]	Qualitative and quantitative analysis of patient neglect at mid-Staffordshire NHS Foundation Trust. The report collected data through interviews with patients and staff	3	Not documenting/reporting patient injuries; Not providing appropriate nutrition or hydration; Patients left in unsanitary/unhygienic conditions; Ward and bedside areas left unclean; Not appropriately considering patient readiness for discharge; Failures to observe, monitor, or record patient data; Unnecessary delays to patient diagnosis	Failures to respond to patient requests to go to the toilet; Not responding to patient’ requests; Not acknowledging concern provided by patients/families; Not providing appropriate assistance for eating and drinking; Failing to listen families; Rudeness Lack of empathy	High workloads and low staffing levels; Protocol breaches; Poor ward management; Communication errors between healthcare staff	Poor safety culture; Lack of training for managing complex patients; Perceived focus on task-focussed rather than patient centred-care; Lack of staff trust in management, fear for jobs, and low morale; Management ignoring staff warnings on care/workloads; Poor organisational leadership
12. Care Quality Commission, 2011 [43]	Inspections of 100 acute hospitals in England in order to assess standards of dignity and nutrition for elderly patients. Methods involved observations, and interviews with staff and patients	3	Not providing appropriate nutrition or hydration; Patients left in unsanitary/unhygienic conditions; Not ensuring patients have call bells in reach	Not providing appropriate assistance for eating and drinking; Failing to help patients to go to the toilet; Not responding to patient’ requests	High workloads and low staffing levels; Failures in identifying poor care; Protocol breaches; Lack of appropriate training for staff	Perceived focus on task-focussed rather than patient centred-care; Poor safety culture; Lack of organisational leadership on priorities of care
13. UK Parliamentary and Health Ombudsman., 2011 [45]	Qualitative investigations (e.g. interviews and document analyses) into the death of ten elderly NHS patients	3	Not providing/monitoring appropriate nutrition or hydration; Not appropriately planning pain management; Ward and bedside areas left unclean; Not appropriately considering patient readiness for discharge; Not documenting/reporting patient injuries	Not providing appropriate assistance for eating and drinking; Not responding to patient’ requests for help; Not aiding patients who are unable to speak; Informing patients of terminal diagnoses in public wards; Not involving families in end-of-life decision-making	Protocol breaches; Communication errors between healthcare staff; Lack of appropriate training for staff; Poor English of caregivers	Perceived focus on task-focussed rather than patient centred-care; Poor safety culture
14. Zhang et al., 2011 [37]	Survey of 414 family members with relatives in US nursing homes. Measures focussed on observed incidences of patient neglect	2-	Not turning patients in bed; Not providing appropriate nutrition or hydration; Not ensuring patient hygiene	Not responding to patient’ requests for help	Patient complexity; Errors in noticing deficiencies in patient care	

The following data extraction exercise was performed. First, the meaning of neglect was reviewed in each paper. Second, behaviours identified in studies of patient neglect were identified. Third, frequencies of neglectful behaviours reported by healthcare staff, patients, and families were captured. Fourth, causal factors identified by articles and reports as contributing to instances of patient neglect were extracted. This included the capture of both qualitative data (TR) and quantitative data (AG). The extracted data were not amenable to meta-analysis due to a mixture of qualitative and quantitative studies being identified. Consistent with similar reviews of literature with mixed forms of data, a narrative analysis was used to synthesise the findings of the review [30,31].

## Results

Figure 1 reports the results of the literature review. Ten research articles were included, with data largely collected in Scandinavia, South Africa, and the US. The majority of articles used survey methods to measure staff, family, or patient observations of neglectful behaviours [32-39]. Two qualitative papers investigated staff perceptions of patient neglect [40,41], and patient perceptions of neglectful behaviours were also of interest [39,42]. Several studies were conducted in elderly care. The hand search identified four qualitative UK government reports investigating patient neglect at both individual and unit/hospital level [24,43-45]. Many discussion articles (e.g. on legal issues) and studies of related topics (e.g. patient dignity, ethics) were also identified, and were informative in understanding what is meant by patient neglect. However, they were not included in the review due a lack of relevant primary data focussing explicitly on patient neglect. The number of studies and reports seems to be increasing rapidly, with 8/14 (57%) being published between 2009–2012.

In comparison to the other literatures linking behaviours and outcomes in healthcare (e.g. medical error) [46], the number of studies investigating neglect is limited, and data was mostly descriptive. Quantitative investigations tended to be cross-sectional survey studies of staff and patients on how often patient neglect occurred in care institutions. These were relatively narrow in scope (e.g. observations of whether healthcare staff fail to wash patients), and did not provide outcome data. In terms of research quality, these studies generally scored 2- according to the SIGN guidelines [29], as their data were not causal and had quite a high probability of bias (e.g. likely social desirability effects in being questioned on neglectful behaviours). Nurses tended to report on instances of other staff showing neglectful behaviours, and patients and families on observations of patients being neglected. Although clinical outcomes of patient neglect were cited as bedsores, malnutrition, infections, dehydration, contractures, and early mortality, no study systematically linked them to behaviour [37]. Furthermore, causal factors underlying perceptions of neglect were not statistically associated, and were discussed in the generality. For example, failures to feed patients were repeatedly cited as being examples of patient neglect, but it was not clear as to whether these occurred due to external system failures (e.g. catering problems), workloads (e.g. too many patients to manage), crises (e.g. diverting attention), team failures (e.g. confusions of responsibility), unintentional error (e.g. incorrect beliefs on patient diet), or a lack of a caring attitude towards patients.

In the qualitative studies and four government reports, specific patient encounters and failings in care were described. In terms of research quality, these studies generally scored 3 according to the SIGN guidelines [29], as their data were derived from case reports and interviews with staff, patients, and families. These often described extreme neglect (e.g. malnutrition contributing to patient mortality), and tended to focus on clear procedural breaches by staff (e.g. pain management protocols). They also focussed on the perspectives of patients and families on why neglect occurred (and feelings of not being cared for), however these were often general and not linked to specific behaviours. As indicated above, there was a lack of clarity for what the research publications meant by ‘patient neglect’. The following section considers further the meaning of patient neglect in order to fully distinguish it from concepts such as error, and to reflect its apparent subjectivity.

### The meaning of ‘patient neglect’

Although patient neglect is a term used by the public and media to describe poor patient care, its clinical, legal, and social meaning appears somewhat unclear. Descriptions of patient neglect in the literature review often invoked implicitly or explicitly Lachs and Pillemer’s [1] (p.437) definition of neglect as “the failure of a designated care giver to meet the needs of a dependent”. Several studies quoted or used a variant of this definition [32,35,37,39,47], or simply referred to neglect as depriving patients of their most basic needs [24,45]. Others studies did not provide a definition, but referred to behaviours broadly in-line with Lachs and Pillemer [1] (i.e. not meeting the needs of a dependent) [34,36,40,41,43,44]. These are consistent with how the UK government describes neglect in the care of vulnerable people (e.g. ignoring the medical or physical needs of patients) [48], descriptions of medical/clinical negligence (a breach of a “duty of care in failing to reach the standard of care required by law”, p.193) [49], or the World Health Organisation’s [50] definition of neglect (“The absence of minimal services or resources to meet basic needs”, p.129). Yet the above definitions are problematic as they focus on the outcome of neglect (e.g. patient malnutrition) without explaining why neglect has occurred (e.g. error, carelessness, intentional abuse). This creates conceptual overlaps with other constructs used to describe poor care, and indicates a need for conceptual refinement in order to aid the design of interventions to focus specifically on reducing instances of neglect.

First, research papers on neglect often discuss patient neglect and patient abuse interchangeably due to their apparent similarity [41]. The literature on ‘patient abuse’ investigates purposeful attempts by healthcare staff to inflict physical or emotional harm (e.g. withholding food) on patients [47]. Although this produces outcomes similar to patient neglect, the workplace psychology literature indicates neglect and abuse at work to be two distinct forms of behaviour. Abuse refers to active attempts by employees to cause harm (e.g. malicious behaviour), whereas neglect refers to passive omissions (e.g. laziness) by employees to ensure a “minimal quality and quantity of work” [51] (p.333). As revealed in an a recent investigation of patients being physically abused in a UK private hospital, there is growing concern over better safeguarding patients from malicious behaviour [52]. Behaviours characteristic of patient neglect (e.g. not feeding a patient) may form part of a pattern of abuse. However if such instances are not a deliberate attempt to harm a patient (e.g. carelessness in not helping a patient to cut-up their food), a distinction must be made between neglect and abuse due to them having psychologically distinct motivational underpinnings.

Second, studies of patient neglect often do not distinguish between neglectful behaviours (e.g. ignoring patients) that have occurred due to poor attitudes on the part of staff, or error [32,35]. A substantial literature exists on medical error [27], and poor care that occurs due to genuine mistakes (e.g. caused by system factors) is considered erroneous and unintentional, and does not represent gross carelessness or a lack of compassion or competence. This distinction is essential, as the causal factors and mechanisms leading to an outcome (e.g. not washing a patient) differ if poor care has occurred due to error (e.g. a communication problem, lack of training) rather than neglect (e.g. staff avoiding the task). In reflecting upon the Mid-Staffordshire scandal, Alghrani and colleagues [21] demonstrate the importance of this distinction. Although UK ‘medical negligence’ laws are intended to punish cases where patients experience injury through professionals failing to meet their duty of care to patients [49], to date the most severe instances of patient neglect at Mid-Staffordshire remain unpunished. This is, in part, due to the difficulties in associating harm caused by patient neglect (e.g. infections due to poor hygiene) with the specific behaviours of individual staff members, and also explaining why those behaviours occurred (e.g. error, staff attitudes, institutional failures institutional failures) [24]. Furthermore, instances of patient neglect (e.g. not displaying compassion) may not violate institutional rules [41], or may be a product of organisational decisions (e.g. budget cuts reducing time spent with patients).

Third, initial inspection of the research articles indicated neglect to be quite a subjective construct, and this partially explains why neglect and error can become confused. For example, qualitative studies of patient neglect frequently take the perspectives of both carers and patients in trying to understand why poor care occurs. Whilst clinical indicators of neglect may be present (e.g. a lack of adequate pain relief), the role of individual caregivers is contested. For example, patients and families may believe neglect has occurred due to healthcare staff simply not caring about them [45]. However, healthcare staff may believe poor care has occurred due to system factors (e.g. workloads that cannot be fulfilled) beyond their control [44]. Furthermore, patient neglect can refer to failures in managing the psychological well-being of patients (e.g. not showing compassion or maintaining the dignity of patients), with no immediate physical harm. Healthcare staff and patients may also have different perspectives as to whether and why such events have occurred [37], what concepts such as ‘dignity’ and ‘compassion’ mean, and their impact upon patient well-being. In comparison to clinical outcomes, the subjective aspects of patient neglect (e.g. failures to demonstrate compassion) are difficult to measure or regulate [53], and complex to reward and prioritise [54]. Yet despite their subjectivity, the interactions between healthcare staff and patients are consistently cited by patients as fundamental to good care [55-57], and are therefore essential for understanding why patient neglect occurs.

#### Re-conceptualising patient neglect

Taking into account the discussion above, and growing public interest in the topic of patient neglect, we propose that it is necessary to further refine the concept of patient neglect, and to differentiate between ‘procedure neglect’ and ‘caring neglect’. This observation was supported by our initial attempts at data extraction, where we found some of the behaviours classified as ‘patient neglect’ to be objectively indicative of poor care, but potentially caused by a range of factors, including error. However, other behaviours classified as ‘patient neglect’ appeared ambiguous and difficult to measure (e.g. not showing compassion), but less easily explained by error.

*Procedure neglect refers to failings in care that fall short of objective and observable institutional and professional standards (e.g. protocols, and regulations).* For example, failing to feed, hydrate, turn or clean a bed-bound patient are instances of procedure neglect. The causes might be due to staff not being inclined to care, or other factors such as system failings and error. Procedure neglect is ‘system-indicated’, in the sense that it is defined by a violation of an institutional procedure or standard. It is focused on behaviours which can be objectively measured, and not perceptions of the attitudes ‘behind’ the behaviours, or patient assessments of the quality of their care (i.e. it is externally assessed).

*Caring neglect refers to failings in care that are below the threshold of being proceduralised (and are unlikely to cause immediate harm), yet lead patients, family and the public to believe that staff are unconcerned about the emotional and physical wellbeing of patients.* This pertains to patient perspectives (or those of families and other caregivers), and specifically attributions about staff being uncaring. Caring neglect might include not being helped to eat, not being treated with dignity and respect, or having concerns dismissed. None of these behaviours are likely to violate a regulation or protocol, nevertheless, patients may see them as indicators of caring neglect.

Procedure neglect and caring neglect are not mutually exclusive. For example, long-term caring neglect can become procedure neglect (e.g., repeatedly neglecting to help feed a patient will result in harm), and if patients or family are aware of the violation of a procedure they may take it as indicative of caring neglect. But, the concepts also diverge. Caring neglect includes behaviours that fall below the threshold of being proceduralised (e.g. monitored using objective metrics), but are important to patients and the quality of their care (e.g., ignoring a request for a glass of water, dismissing a complaint of pain). However, procedure neglect can be invisible to patients (e.g., incomplete patient notes) and thus cannot lead to patients perceiving caring neglect. Indeed, some instances of procedure violation may be taken by patients to indicate caring (e.g., nursing staff allow patient relatives to break visiting hours rules). The key difference is that procedure neglect is assessed from an institutional standpoint while caring neglect is assessed from a patient standpoint. Perhaps most critically, instances of caring neglect may provide an early warning sign to procedural failings that will eventually harm patients, and are typically assessed after harm has occurred (e.g. through auditing or retrospective case review).

Public concern with patient neglect can focus on the neglect of institutional procedures, for example failures in washing patients or documenting data, and these incidents can occur due to a variety of reasons (e.g. error, a lack of a caring attitude). However, significant concerns also focuses on the attitudes and orientation of staff that are attributed through instances of caring neglect (e.g. ignoring a patient, rudeness, failing to respond to seemingly minor requests) that violate public expectations of ‘being cared for’. These behaviours are often below the threshold of institutional monitoring (and therefore are not instances of procedure neglect), are subjective, and are highly salient to patients as indexes of staff attitude and quality of care. While abuse, error and procedure neglect are clearly defined, caring neglect is often contested (e.g. patients may believe staff do not care about them, when this is not the case), and there may be divergent explanations as to whether and why a behaviour indicative of an uncaring attitude has occurred [58]. Regardless of this, where patients do perceive caring neglect, it can be a visceral aspect of their care [55] with inaccurate perceptions impacting upon their emotional well-being and satisfaction with treatment [59-62].

In order to better identify the causes of procedure and caring neglect, we also separate between ‘proximal causes’ and ‘distal causes’ of neglect. Our initial data extraction exercise to identify the causes of patient neglect found a mixture of ‘on-the-ground’ and ‘latent organisational’ factors to cause patient neglect. Proximal (i.e. on-the-ground) causes of neglect include factors such as high-workloads, poor ward leadership, or negative attitudes towards patients [41]. They often emerge from distal causes (i.e. latent institutional problems), such as poor hospital management or institutional change [24]. Distinguishing between these factors is essential for understanding the ‘root causes’ of patient neglect, and is consistent with organisational psychology theory on the causes of organisational failure [63].

Using the conceptual framework outlined above to guide the data extraction process, the findings of the literature review are presented in the sections below. They are summarised in Table 1.

### Behaviours identified in studies of patient neglect

The reviewed articles identified a wide range of behaviours associated with patient neglect. These are listed according to study in Table 1 and conceptualized in terms of caring and procedure neglect.

For procedure neglect, studies tended to collect data either through surveys with patients and caregivers, or through retrospective analysis of extreme cases of patient neglect (which involved primarily patient input). The behaviours highlighted in these studies consist of three types. First, neglecting the maintenance of a patient’s physical condition, for example not washing patients, feeding patients, or turning patients over in bed [32,37]. Second, neglecting aspects of a patient’s treatment, for example planning patient discharges, documenting patient injuries, planning pain management, delaying diagnoses, and monitoring patients [44,45]. Third, neglecting elements of ward care, for example not ensuring wards and bed spaces are clean [24]. As would be expected, types of neglectful behaviours were associated with job roles. For example, neglectful behaviours by medical staff related to behaviours such as ignoring patient perspectives in decision-making, or not planning pain management adequately.

In terms of caring neglect, surveys and interviews with healthcare staff on their observations (and in some cases participation) of neglect highlighted being rude to patients, not responding to patient complaints of pain, purposefully delaying help for patients, intentionally ignoring patients, avoiding contact with patients, preferring to socialise with colleagues than treat patients, and prioritising some patients over other others due to liking them more [32,34,35,38,41]. Surveys and interviews with patients and families also recurrently highlighted behaviours associated with caring neglect, and these often focused on failing to provide emotional support [36,37,42,44]. Examples included behaviours such as not being listened to in decision-making, not being shown empathy or compassion when in need, failing to communicate properly, rudeness, a lack of urgency for providing help, and a belief that staff are unwilling to give assistance with basic tasks such as eating, drinking, speaking, and going to the toilet.

### Discrepancies in staff and patient perceptions of patient neglect

Where possible, the reported frequency of neglectful behaviours between healthcare staff, patients, and families were compared (as reported in Table 1). Discrepancies were identified between what nurses reported about their own neglectful behaviours, their observations of other nurses, and the reports of patients and their family members. There was a persistent tendency for nurses to observe higher incidences of other nurses engaging in neglectful behaviours compared to reports on their own behaviour. These differences were particularly marked for not changing linen or underwear (18% to 8%), using sedatives to minimise workload (31% to 8%), failing to wash patients (39% to 20%), ignoring patients (66% to 44%), and not changing diapers (42% to 21%). The lower rates of self-reported behaviour could be due to nurses not wanting to admit to being neglectful, failing to see their own behaviour as neglectful, or because there are a few neglectful nurses whom most nurses have observed.

Patients and their family members frequently reported the highest incidents of neglectful behaviour. Swahnberg et al. [36] found 73% of patients had, over their lifetime, experienced staff disobeying ‘ethical principles’. Research in geriatric care [37] shows that 21% of family members with a relative in a nursing home had observed neglect “such as failure to rotate or flip this person to prevent bed sores, failure to provide a person with food, water, shelter, hygiene, medicine, comfort, or personal safety or ignoring request for help” during the preceding 12 months (p.65). These rates are high given that patients and family members would have little exposure to the health services compared to, for example, the nurses who have much better opportunity to observe neglect on a daily basis.

In understanding what neglect is, and why it occurs, these divergences of perspective are also important. Specifically, there seems to be a tendency for patients and family to focus on caring neglect [36,41] while health care staff focus on procedure neglect [32,35]. Moreover, the perception of the causes also seems to vary. Research with nursing staff shows that nurses often feel neglect occurs due to an inability to meet the demands placed upon them [40]. Frequently, staff feel overwhelmed as they do not have the resources or training to provide optimal patient care, and may be functioning within a system within which problems already exist [40,44]. For example, failures to ensure an appropriate pain management programme can result from external pressures that result in constant delay, administrative failures elsewhere in the system, a misunderstanding or lack of clarity on patient condition, uncertainty, lack of training, and failures in team communication. Yet, from the perspective of patients and families (and possibly other staff members), neglect may appear to occur due to a lack of care or incompetence [45].

### Causal factors identified as leading to patient neglect

A variety of causal factors were identified by articles and reports as contributing to instances of patient neglect. None were systematically associated with poor care (so cannot be linked directly to ‘procedure’ or ‘caring’ neglect), and thus they offer fruitful avenues for future research. Consistent with the themes of ‘proximal’ and ‘distal’ causes of patient neglect, Table 1 lists contributory factors leading to patient neglect by study. Proximal causes of patient neglect tend to refer to the ‘on-the-ground’ aspects of care that lead to patient neglect, including workloads, staff burnout, teamwork and relationship between staff and patients. The distal causes refer to those causes which create the condition for patient neglect to occur, including organisational management, safety culture and systems for reporting poor care.

#### Proximal causes of patient neglect

The review found high workloads to be the most commonly identified cause of patient neglect [32,37,41,43]. Frequently, they led to neglect through creating situations where staff did not have time to engage in either ‘caring’ behaviours (e.g. listening to patients, responding quickly to their requests) or fulfil expected tasks (e.g. washing and cleaning patients). This is consistent with research showing nurses to ration healthcare activities according to system demands [64]. High workloads generally refer to under-resourcing (e.g. personnel or materials) which results in too many patients to care for, too many tasks to perform, constantly changing workloads, and burgeoning bureaucracy. Training is essential for managing such workloads, and lack of staff training is also cited as leading to patient neglect [43,44]. Healthcare staff were found to be placed in positions of care for which they had not received appropriate instruction (e.g. for managing complex patients), and were left vulnerable to providing poor-quality care. Alongside constraining the ability of staff to meet work demands, research shows high workloads also shape how healthcare providers think about risk. Consistent with investigations on rule-breaking and workplace violations [65], high workloads result in staff constantly shifting their beliefs on the acceptability of risk and quality of care so that they can manage their workloads [66]. Tasks and rules are sacrificed in order that a limited range of care objectives can be met. These can mean that compromises are made in care, heightening the likelihood of caring or procedure neglect.

Staff burnout was also indicated to underlie instances of patient neglect [32,35], and although closely related to workload (staff burnout frequently occurs due to overwhelming demands at work) the psychological mechanisms underlying neglect appear different [32,37]. Burnout describes a state of mental exhaustion arising from an inability to meet the demands of a work setting [67], and it results in negative attitudes, emotions and behaviours towards one’s work [68]. Medics and nurses generally suffer high levels of burnout, and this is caused by high patient-to-nurse ratios, workload, conflict, emotional demands, job insecurity, and low job satisfaction [67,69-73]. Burnout sufferers experience depression, physical illness, and poor work performance, and *emotional exhaustion* and *depersonalisation*. These can result in detached and cynical attitudes, and a lack of empathy or compassion. Research on ‘compassion fatigue’ identifies a variety of organisational and individual level factors that can produce feelings of burnout [74]. In terms of patient neglect, burnout is likely to result in a reducing of ability to empathise and demonstrate compassion to patients, and to complete demanding tasks and high workloads [75]. This is consistent with research demonstrating the link between correlates of burnout (e.g. stress) and counterproductive [76].

Problems in multidisciplinary teamwork (e.g. failures in communication, or providing appropriate assistance) were also found to lead to patient neglect [43,44]. As demonstrated in the medical error literature, effective teamwork is essential for sharing work duties and coordinating activities fundamental to patient care [77]. The literature review highlighted instances of patient neglect that occurred through communication errors between staff, or shared failures to notice or take action on irregular or problematic aspects of patient care [37,44,45]. Patient safety research shows organisational and team leadership is particularly important for establishing norms of behaviour and quality of care [78], and a failure of team leaders to set appropriate standards for multidisciplinary healthcare teams often underlies substandard care [41,43]. Whilst research on neglect has not focussed on leadership, research has demonstrated the importance of effective medical, nursing and managerial leadership (e.g. providing clarity on goals, encouraging open communication, creating opportunities for improving care) for ensuring quality of care and patient satisfaction [79-81]. Thus it is of considerable importance for understanding the occurrence of patient neglect.

Finally, relationships between healthcare staff and patients were also found as a factor shaping procedure and caring neglect. For example, research in South Africa shows high workloads, conflicts with patients, and minimal support from management to result in nurses developing beliefs on the need to assert control over patients, and to do this through rationing care (both in terms of attitudes towards patients, and supporting their physical well-being) [41]. In these conditions, patients can be categorised as ‘good’ or ‘bad’, and treatment is commensurate with this status [34]. In some cases, healthcare staff can feel frightened of patients, resulting in them avoiding interactions and care encounters wherever possible [32]. Such research is consistent with investigations on the extent to which attitudes such as compassion towards patients can be discretionary and visible [82]. From the perspective of patients, they can feel neglected when healthcare staff do not take into account their concerns when making decisions, and can be afraid to raise concerns with their care (allowing poor care to continue) for fear of being punished [42]. They also can feel unable to discuss aspects of healthcare quality outside of their own treatment [83]. Furthermore, they can believe that neglect occurs due to staff not adhering to ethical codes of practice for engaging them in decision-making and care [36].

#### Distal causes of patient neglect

An important distal cause of patient neglect is organisational management. In the mid-Staffordshire [24], job insecurity, lack of resources, poorly managed change, and incoherent management created conditions for neglect to occur. High workloads, stress and poor leadership resulted in staff being constrained in their ability to provide good care, alongside creating widespread de-motivation, burnout, and disengagement [24,47]. This is consistent with research showing that healthcare staff in poor organisational environments report lower job satisfaction [84], frustration, and disengagement [85,86], and lower quality of patient care [73]. Thus, as in the case of Mid-Staffordshire, a lack of organisational support/stability compromises patient care through making it difficult for employees to understand or meet expected standards, alongside reducing their willingness to perform to a high standard. This reflects healthcare research linking job satisfaction to poor performance [87], and positive beliefs about management to greater employee commitment [88]. Furthermore, several studies in the review highlighted how management prioritisation of particular targets contributed to instances of patient neglect [43,45]. Specifically, through leadership focusing and rewarding the completion of goals and tasks over ‘caring’ activities, healthcare providers are pushed towards having ‘tunnel vision’ [89] for completing tasks that are clearly rewarded. This de-prioritises activities not formally measured or rewarded, but important for preventing procedure and caring neglect.

Poor safety culture is also a distal casual factor in patient neglect [24,45]. Many of the characteristics symbolic of poor safety culture (e.g. lack of resources, poor management, constant change, unclear performance standards [90] are found in studies of patient neglect, with care often being task-centric rather than patient-centric (e.g. having staff focus on bureaucratic targets) [24,43]. Furthermore, in some cases, a lack of ‘*psychological safety’* for staff openly discussing errors, rule-violations, or poor care can lead to poor patient care not being identified and ameliorated [24,41,91]. Consistent with safety culture theory, this lack of openness symbolises the prioritisation of safety by management, and in-turn influences the behavioural norms relating to care provision [92]. For example, healthcare research shows staff attitudes towards risk and safety-related tasks in theatre predict rule compliance and the following of safety protocols [93,94] and medical and nursing staff perceptions of safety climate predict patient safety and patient satisfaction [95,96]. In terms of patient neglect, poor safety culture is likely to symbolise the importance placed by the healthcare institutions on activities related to caring and following procedures, and the importance of preventing neglect.

Inadequate systems for reporting patient neglect are also identified as being casual factors in instances of poor care [21]. Organisational psychology research has long highlighted the importance of reporting systems for identifying ‘symptoms’ of organisational failure before they become widespread or serious [63]. As with medical error, it is likely that staff being able to report on observations or fears on substandard care is essential for avoiding systemic neglect within a healthcare system [63]. Failures to ‘whistle-blow’ (or listen to whistle-blowers) will result in poor care not being identified or learnt from [21,63,97,98]. Yet incident reporting on safety incidents within healthcare remains low, with only 10% of safety incidents being captured [99]. This is likely to contribute to a culture where poor care is not discussed or investigated [66]. For example, nearly a quarter of UK doctors have reported working with an impaired or incompetent doctor, yet were unable to report this to an appropriate body [100]. Barriers to staff reporting [98] include i) believing that the organisation does not really want reports on poor care, ii) believing that reporting systems are not reliable or fair, and iii) fear of repercussion. For patient neglect, this lack of reporting makes it difficult for the organisation to identify poor care before it becomes systematic or extreme.

## Discussion

There is growing public concern over patient neglect in healthcare institutions. To understand and explain what patient neglect is, and why it occurs, it has been necessary to draw on a range of psychology literatures. These include the error and workplace deviance literatures [51,63,65], the patient safety, organisational psychology, and non-technical skills literatures [23,27,101], and social psychological research on perspective taking, burnout, and caring [55,59,61,68]. Using these literatures, we have conceptualized patient neglect as clearly distinct from error (purposeful actions with unintentional consequences) and abuse (purposeful attempts to inflict physical or emotional harm). In order to contribute to public dialogue and academic analysis, we have differentiated between caring and procedure neglect. Whilst procedure neglect refers to the objective measures of patient neglect (e.g. not feeding patients) that might result from error, abuse, or lack of caring, caring neglect attempts to capture the subjective and emotional aspects of poor care. Caring neglect can be damaging to the emotional well-being of patients (e.g. dignity) even if subjective beliefs about staff attitudes are not accurate. The concept of caring neglect appears important for addressing public concerns because this is often what patients and their families are worried about, yet it is largely invisible to healthcare institutes because it refers to behaviours which have not been (and possibly cannot be) fully proceduralised. Central to caring neglect are the interactions, perceptions, and beliefs of healthcare staff and patients. It relates to behaviours that are physically or emotionally neglectful towards patients, represent a perceived or actual lack of caring and compassion for patients.

**Figure 2:**
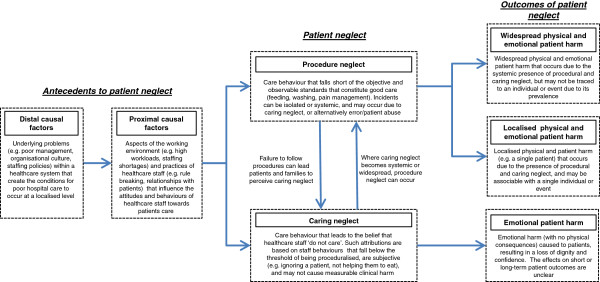
Conceptual model of patient neglect.

Consistent with literatures on accident causation [25,102], a range of proximal factors (e.g. workloads) were found to shape the medical and nursing care provided to patients, and these were in turn influenced by distal (e.g. institutional) factors such as organisational leadership. The review data does not enable us to separate the causal pathways leading to caring neglect from the pathway leading to either error or abuse. However, it does seem that caring neglect can lead to a distinctive negative outcome, namely, emotional patient harm from loss of dignity or feeling uncared for. Crucially for developing interventions, patient neglect is found to occur due to both factors relating to staff attitudes, and others factors relating to such as poor communication training, or systems (overlapping with the medical error literature). Figure 2 presents an initial psychological model of patient neglect. It illustrates the findings of the review to explain why neglect occurs, and considers the potential outcomes of neglect. These require further study, and future modelling should consider the role of error in understanding why neglect occurs.

Only ten research articles were identified as reporting primary data on patient neglect in healthcare settings. Few academic articles were published in the UK, despite the public concern. This may reflect i) a dearth of research into the subject, ii) the use of inappropriate search terms, or iii) that research investigating patient neglect is obscured within other studies focussing upon error or abuse. However, it is notable that in conceptualising patient neglect, our review referred informally to other key health service research literatures. For example, it considered medical error and patient abuse, both of which can describe behaviours associated with procedure neglect, albeit in the context of mistakes or intentional harm. It also referred to work investigating what patients consider to be ‘good care’. This often relates to interactions between healthcare staff and patients (good technical care is assumed) [56]. Professional, approachable, and compassionate attitudes towards patients, as embodied by caring behaviours, are seen as the bedrock of good care [55,56,103]. Their absence is an element of caring neglect [104], and although research on good care does not focus explicitly on the neglect of patients, it may aid our understanding of patient neglect.

However, current methodologies for studying error (e.g. retrospective record review) or good care (e.g. audits) may struggle to assess why patient neglect occurs, and new research techniques are required (e.g. focussing on complaint data). This is perhaps demonstrated in the ratings of research quality for the publications included in the review [29] – studies were largely assessed as being descriptive and non-predictive. Further work is required to understand the causes of both procedure and caring neglect, and this analysis was restricted to publications in the literature review. Other factors may also be important, including clinical training, individual differences (personality, motivation), socio-economic factors, and expectations on care. Furthermore, the international nature of the research on patient neglect demonstrates wider interest in the topic. This is in turn poses questions about patient neglect in countries with different institutional procedures and/or different patient expectations, and consequently the distinctive steps that might be taken to improve care.

## Conclusions

In this article we developed a social psychology-based conceptual model to explain the occurrence and nature of patient neglect. To date, patient neglect has been difficult to conceptualize because it has one component which is defined by institutional procedures (procedure neglect) and another component which is defined by patient expectations about care (caring neglect). Separating these components reveals a possible divergence of perspective between medical institutions and the public on the nature of patient neglect. Moreover, it leads to new questions: Can the behaviours which lead patients to feel neglected become proceduralised or measured? And if not, then how can one ensure that staff engage in the multitude of non-measurable behaviours which lead patients to feel cared for? This is a critical question for patient safety specialists and policy makers, as it indicates regulations and targets are unlikely to reduce the caring neglect that is often the focus of patient complaints [11]. Indeed, creating rules that reduce the ability of healthcare staff to engage in discretionary caring acts (e.g. filling forms on caring) may aggravate the problem. To begin to address these questions, future research must i) further investigate healthcare staff and patient perceptions of patient neglect, ii) systematically investigate the relationship between caring neglect and procedure neglect and their impact upon health outcomes, and iii) consider the appropriateness of current patient safety interventions for reducing patient neglect.

## Competing interests

The authors declare that they have no competing interests.

## Authors’ contributions

Conceptualisation of publication and of procedure and caring neglect, literature search, extraction of qualitative data, data analysis, analysis of causal factors underlying neglect, development of conceptual model and data presentation (TR). Extraction of quantitative data, conceptualisation of procedure and caring neglect, comparison with other patient safety concepts, data analysis, development of conceptual model and data presentation (AG). Both author read and approved the final manuscript.

## Pre-publication history

The pre-publication history for this paper can be accessed here:

http://www.biomedcentral.com/1472-6963/13/156/prepub
